# Electrical Properties of Model Lipid Membranes

**DOI:** 10.3390/membranes12020248

**Published:** 2022-02-21

**Authors:** Monika Naumowicz

**Affiliations:** Department of Physical Chemistry, Faculty of Chemistry, University of Bialystok, K. Ciolkowskiego 1K, 15-245 Bialystok, Poland; monikan@uwb.edu.pl

Biological membranes are essential components of the living systems, and processes occurring with their participation are related mainly to electric phenomena such as signal transduction, existence of membrane potentials, and transport through the membrane. It is well known that the universal model of cell-membrane structure is the lipid bilayer, which constitutes the environment for integral and surface membrane proteins. Thus, much attention has been given to the study of the organization and properties of these structures concerning both experimental and theoretical aspects. As systematic examinations are impeded by the complexity of the natural membranes, the best approach to conduct detailed physical and chemical studies of biological membranes is to use simplified well-defined model lipid membranes. These models can be used to obtain information on the properties of real biological membranes and associated electrochemical reactions. Among the most commonly used are liposomes, planar lipid membranes, membranes on solid substrates, and lipid monolayers on the free surface.

This *Membranes* Special Issue discusses the significant recent progress that has been made recently in the study of the electrical properties of various model membrane systems used for mimicking biological membranes. The Special Issue contains eight articles: seven research articles and one review. The complete description of each study and the main results are presented in more detail in the full manuscript, which the reader is invited to read. A summary of the articles is presented here.

The first research article of this Special Issue by Dziubak et al. [1] describes a novel approach to fabricate supported membranes on Au electrodes functionalized with thioglucose, using bicelles as precursors. For characterization the authors employed surface-enhanced infrared absorption spectroscopy, atomic force microscopy, and electrochemical methods, which are customary in the field. They indicate that more uniform and stable lipid membranes may be obtained by adopting a freezing–thawing approach. The presented approach offers an alternative way to obtain stable planar lipid membranes with good insulating properties. In perspective, the benefit of such an approach is that bicelles are known to be a suitable lipid environment for the reconstitution and studies of the transmembrane proteins. Therefore, more complex cell membrane mimics can be obtained by depositing bicellar mixtures on electrodes.

The article of Naumowicz et al. [2] is a continuation of research on the interaction of model membranes of increasing complexity with naturally occurring phenolic compounds. Electrophoretic light scattering and impedance spectroscopy were applied to study the effects of cinnamic (CinA), *p*-coumaric (*p*-CoA) and ferulic (FA) acids on electrical properties of spherical bilayers and small unilamellar vesicles. It was found that at acidic pH all tested compounds were able to solubilize into the membrane and permeate it. However, at neutral and alkaline pH, the CinA could be partially inserted into the bilayers, whereas *p*-CoA and FA could be anchored at the membrane surface. Since intracellular penetration seems to be a key determinant of drug action, the obtained results suggest that electrochemical methods can be employed for predicting pharmacological activity and bioavailability of phenolic acids.

The article of Chachaj-Brekiesz et al. [3] reports a study of the surface potential of Langmuir monolayers formed by the most abundant phospholipids of mammalian cell membranes. In this context, the electric surface potential, surface pressure isotherms and the compression moduli were recorded, and the areas per headgroup and the dipole moment were calculated. The authors correlated the results obtained from the density functional theory modeling with experimentally determined values using multiple linear regression and proposed an improved protocol for estimating individual contributions to a three-layer capacitor model. Their methodology can be applied for other phosphatidylcholine derivatives with different hydrocarbon chain lengths and saturation but similar hydration. The article is an interesting contribution to the field of membrane biophysics, and the idea of performing a systemic study on this topic is great as the literature reports on different values due to the experimental settings. 

The article authored by Meleleo [4] explores the ability of the natural drug resveratrol to penetrate planar lipid membranes of varying composition and represents another valuable contribution to the field of drug–lipid interactions in this Special Issue. Resveratrol is a polyphenolic molecule which is believed to incorporate into the lipid bilayer similar to cholesterol. The main outcome of the performed electrophysiological measurements was that resveratrol incorporates into the zwitterionic and neutral membranes and forms transient conductive units, whereas it is unable to interact with the negatively charged membranes. The results obtained by simultaneously monitoring membrane conductance and capacitance may help elucidate the mechanism of action by which resveratrol exerts its beneficial effects on human health, such as its antioxidant or anti-microbial properties and its lipid radical scavenging.

In the article of Petelska et al. [5], it was shown, on the basis of both experimental and theoretical data, that the electrical properties of blood cell membranes are altered after fatal alcohol poisoning. Erythrocytes and thrombocytes have a relatively simple structure. They are therefore ideal cellular models for studying changes in the physicochemical properties of membranes under the influence of small amphiphilic solutes, such as ethanol. It has been suggested that in cellular systems the toxicity of ethanol is due to its interaction with membranes. Due to the lack of literature data on the effect of fatal poisoning with ethyl alcohol on the equilibria between the membranes of erythrocytes and thrombocytes and the surrounding environment, the authorts successfully applied a mathematical model to describe these equilibria. 

In their original research article, Maček Lebar et al. [6] explore the electroporation properties of planar lipid bilayers with different compositions. The bilayers were formed using two types of lipid molecules: 1-pamitoyl-2-oleoyl phosphatidylcholine (POPC), lipid molecules with a zwitterionic head group and almost zero spontaneous curvature, negatively charged 1-pamitoyl-2-oleoyl phosphatidylserine (POPS) molecules with negative spontaneous curvature, and a mixture of both lipid types in a 1:1 ratio. The authors were successful at showing the dependence of capacitance, breakdown voltage, rupture time or radii or pores with membrane type. They pointed out that water pores form easily in more disordered planar lipid bilayers, and the radii of these pores are smaller than in more ordered bilayers. In the case of lipid mixtures, water pores form even more easily, probably because of smaller disordered domains in the bilayer and/or weaker regions at the domain boundaries.

The paper by Vitkova et al. [7] deals with the study of alteration of dielectric properties, the degree of hydration, the rotational order parameter and dipole potential of lipid bilayers in the presence of simple carbohydrates. Frequency-dependent deformation of cell-size unilamellar lipid vesicles in an alternating electric field and fast Fourier transform electrochemical impedance spectroscopy were used to measure the specific capacitance of phosphatidylcholine lipid bilayers in aqueous solutions of sucrose, glucose and fructose. The results show that some small carbohydrates are able to alter the dielectric properties of membranes, their structure, and the order related to membrane homeostasis. The reported data are also relevant for future studies on the response of lipid bilayers to external physical stimuli such as electric fields or temperature changes.

In the review [8], Raval et al. highlight the importance of the surface topography of nanomaterials and the role it plays in their interactions with biological materials, using TiO_2_ as an example. They also analyze the interplay of the elastic and adhesive contributions of lipid vesicles to adsorption on these solid surfaces, using numerically predicted lipid vesicle shapes. Finally, they explore the origin of electrostatic interactions between lipid bilayers and charged solid surfaces using established statistical mechanical approaches and show that the electrostatic interaction between the zwitterionic lipid head-groups and the charged solid surface results in a perpendicular orientation of the lipid head-groups, leading to tighter packing of the lipids.

Studies of the electrical properties of model lipid membranes have been carried out for many years. However, there are still many issues that have not been verified experimentally and for which the existing results are incomplete or inconsistent. Therefore, the main objective of this Special Issue was to collect recent scientific and review articles on the electrical properties of model lipid membranes. This objective has been successfully achieved, for which I express heartfelt appreciation to all authors and reviewers for their excellent contributions.

## Data Availability

Not applicable.

## References

[1] Dziubak D., Strzelak K., Sek S. (2020). Electrochemical Properties of Lipid Membranes Self-Assembled from Bicelles. Membranes.

[2] Naumowicz M., Zając M., Kusaczuk M., Gál M., Kotyńska J. (2020). Electrophoretic Light Scattering and Electrochemical Impedance Spectroscopy Studies of Lipid Bilayers Modified by Cinnamic Acid and Its Hydroxyl Derivatives. Membranes.

[3] Chachaj-Brekiesz A., Kobierski J., Wnętrzak A., Dynarowicz-Latka P. (2021). Electrical Properties of Membrane Phospholipids in Langmuir Monolayers. Membranes.

[4] Meleleo D. (2021). Study of Resveratrol’s Interaction with Planar Lipid Models: Insights into Its Location in Lipid Bilayers. Membranes.

[5] Petelska A., Szeremeta M., Kotyńska J., Niemcunowicz-Janica A. (2021). Experimental and Theoretical Approaches to Describing Interactions in Natural Cell Membranes Occurring as a Result of Fatal Alcohol Poisoning. Membranes.

[6] Lebar A.M., Miklavčič D., Kotulska M., Kramar P. (2021). Water Pores in Planar Lipid Bilayers at Fast and Slow Rise of Transmembrane Voltage. Membranes.

[7] Vitkova V., Yordanova V., Staneva G., Petkov O., Stoyanova-Ivanova A., Antonova K., Popkirov G. (2021). Dielectric Properties of Phosphatidylcholine Membranes and the Effect of Sugars. Membranes.

[8] Raval J., Gongadze E., Benčina M., Junkar I., Rawat N., Mesarec L., Kralj-Iglič V., Góźdź W., Iglič A. (2021). Mechanical and Electrical Interaction of Biological Membranes with Nanoparticles and Nanostructured Surfaces. Membranes.

